# Pod-based e-cigarettes versus combustible cigarettes: The impact on peripheral and cerebral vascular function and subjective experiences

**DOI:** 10.18332/tid/162366

**Published:** 2023-05-26

**Authors:** Ziyad Ben Taleb, Danny Dabroy, John Akins, Michael Douglas Nelson, Mohammed Ebrahimi Kalan, Mary Rezk-Hanna, R. Matthew Brothers

**Affiliations:** 1 Department of Kinesiology, College of Nursing and Health Innovation, The University of Texas at Arlington, Arlington, United States; 2 Institute for Exercise and Environmental Medicine, Texas Health Presbyterian Hospital, Dallas, United States; 3 The University of Texas Southwestern Medical Center, Dallas, United States; 4 School of Health Professions, Eastern Virginia Medical School, Norfolk, United States; 5 School of Nursing, University of California, Los Angeles, Los Angeles, United States

**Keywords:** pod-based e-cigarette, cigarettes, vaping, smoking, cardiovascular disease risk

## Abstract

**INTRODUCTION:**

The vaping epidemic in the US has been largely attributed to the emergence of pod-based e-cigarette devices. While these devices continue to be promoted as alternatives to cigarettes, their impact on cardiovascular and behavioral outcomes remains incompletely understood. This study assessed the impact of pod-based e-cigarettes on peripheral and cerebral vascular function, along with subjective experiences among adult cigarette smokers.

**METHODS:**

In a crossover laboratory design study, a total of 19 (e-cigarette naïve) cigarette smokers (aged 21–43 years) attended two lab sessions. In one session, participants smoked a cigarette and in the other, vaped a pod-based e-cigarette. Participants completed questions assessing subjective experiences. Peripheral macrovascular and microvascular function was assessed via brachial artery FMD and reactive hyperemia, while cerebral vascular function was assessed as the blood velocity response of the middle cerebral artery during hypercapnia. Measurements were taken before and after exposure.

**RESULTS:**

Compared with baseline, there was a reduction in peripheral macrovascular function (indexed by FMD), following e-cigarette (pre=9.3±4.3%; post=6.4±4.1%) and cigarette use (pre=10.2±3.7%; post=6.8±3.8%; main effect of time p<0.0001). Cerebral vascular function (indexed by cerebral vasodilatory response during hypercapnia) was also reduced following e-cigarette (pre=53±19%; post=44±15%) and cigarette use (pre=54±21%; post=44±17%; main effect of time p<0.01). The magnitude of reduction in peripheral and cerebral vascular function was similar between conditions (condition × time, p>0.05). Compared with vaping an e-cigarette, participants scored higher for measures of satisfaction, taste, puff liking, and suppression of craving following smoking (p>0.05).

**CONCLUSIONS:**

Similar to smoking, vaping a pod-based e-cigarette leads to an impairment in peripheral and cerebral vascular function while providing a reduced subjective experience compared with a cigarette among adult smokers. While these data challenge the notion that e-cigarette use is a safe and satisfactory alternative to cigarette use, large longitudinal studies are needed to assess the long-term impact of pod-based e-cigarette devices on cardiovascular and behavioral outcomes.

## INTRODUCTION

While cigarette smoking rates have experienced a historic decline in the United States (US), from 21% in 2005 to 12.5% in 2020^1^, the use of other tobacco products has increased^2^. Specifically, the use of electronic cigarettes (e-cigarettes) among US youth has dramatically increased with more than 2 million middle and high school students reporting current use in 2021^3^. Although e-cigarettes are often marketed as a less harmful alternative to combustible cigarettes, e-cigarettes expose users to several chemicals such as nicotine, volatile organic compounds, carbonyls, and particulate matter^4^. Moreover, the notion that e-cigarettes are less harmful than combustible cigarettes may not translate to reduced burden of cardiovascular disease. In particular, the cardiovascular system is extremely sensitive and evidence exists demonstrating a nonlinear dose-response relationship between light smoking (<3 cigarettes/day) and development of cardiovascular disease^5^. Accordingly, even low exposure to harmful constituents in e-cigarettes could negate any claims of harm-reduction^5^.

Unlike combustible cigarettes, research assessing the health effects of e-cigarette use on cardiovascular function is still in infancy^4^, with studies showing that e-cigarette exposure is equally detrimental as combustible cigarettes^6,7^, mildly detrimental^8,9^, or not detrimental to cardiovascular function at all^10,11^. These mixed results are likely attributable to several factors including the use of older generations of e-cigarette devices that deliver lower doses of nicotine concentration compared with combustible cigarettes^5,12^. Moreover, cerebrovascular function has largely been overlooked in studies assessing health effects associated with e-cigarette use, representing a critical gap in the literature. This is particularly important given that reductions in cerebrovascular function is a predictor and contributor to various cerebrovascular events^13^.

Evidence from a recent systematic review highlighted the urgent need for rigorous studies comparing cardiovascular effects of combustible cigarette smoking with the ‘latest generation’ of high-nicotine-delivery pod-based e-cigarettes^14^, and whether these devices could deliver a similar sensory experience to cigarette smoking^15^. The current study directly addresses this knowledge gap by examining the impact of the acute exposure to ‘next generation’ pod-based e-cigarettes on peripheral and cerebral vascular function. Also, as a secondary aim, we will assess subjective experiences following vaping of a pod-based e-cigarette with direct comparison to combustible cigarettes among regular cigarette smokers.

## METHODS

### Participants

A total of 19 participants were recruited from the University of Texas at Arlington and the surrounding area. Participants were healthy cigarette smokers aged 21–43 years (mean=25.2; SD=5.4) who smoked at least 5 cigarettes/day in the last 12 months and were e-cigarette naïve. Participants abstained from smoking for at least 12 hours before each laboratory visit, which was confirmed by exhaled carbon monoxide levels <5 ppm. Participants were asked to abstain from food and caffeinated beverages ≥6 hours prior to the visits. Exclusion criteria were self-reported history of chronic health problems or psychiatric conditions and the regular use of prescription medications (other than vitamins or birth control). Females were excluded if they were pregnant (verified by urinalysis) or breastfeeding.

### Study design and procedure

This is a crossover design laboratory study that was conducted in the Nicotine and Tobacco Research Laboratory at the University of Texas at Arlington. Each participant completed two laboratory visits that were counterbalanced to account for first-order effect. These two laboratory visits differed by study condition (smoking vs vaping) and were separated by a ≥48-hour washout period to avoid carryover effect. Accordingly, participants smoked one combustible cigarette during one visit and vaped a pod-based e-cigarette device (JUUL, tobacco flavor; 5% nicotine) in the other visit. For both conditions (combustible cigarette and e-cigarette), participants followed a standardized puffing protocol, and were instructed by research staff to take 10 puffs (3 seconds each), separated by 30 seconds^16^. This approach ensured that regardless of the tobacco product used, participants had similar levels of exposures and nicotine delivery^16,17^. During each session, participants were seated on a comfortable recliner chair to simulate a natural smoking/vaping experience.

### Outcome measures

*Peripheral vascular function*

This was assessed using flow mediated dilation (FMD) and post-occlusive reactive hyperemia (RH), as previously described^18^. Briefly, a pneumatic cuff connected to a rapid inflation device (Hokanson Model E20 Rapid Cuff Inflator; Bellvue, WA) was placed just distal to the antecubital fossa and the brachial artery was imaged using high-resolution, duplex Doppler ultrasound. An adjustable frequency (10–13 MHz) linear array transducer (LOGIQ P5, GE Healthcare; Chicago, IL) was selected for optimal B-mode signals of the brachial artery and held in a stereotactic clamp 5–10 cm proximal to the antecubital fossa. Once a suitable image was obtained and optimized for clear delineation between the lumen and vessel walls, duplex mode (at a pulsed frequency of 5 MHz) was utilized for the continuous measurement of brachial artery diameter and blood velocity. The sample volume was set to encompass the entire lumen, without extending into the surrounding tissue, at an insonation angle of 60°. All images were recorded using commercially available screen-capture software (Elgato Video Capture, Corsair; Fremont, CA). Each image was analyzed offline using continuous edge-detection software (Cardiovascular Suite, Quipu; Pisa, ITA) along a section of the artery with clearly defined vessel walls, while second-by-second blood velocity was taken as the entire Doppler envelope. Due to technical difficulties/loss of follow up, data from one of the participants was not collected for the e-cigarette condition (n=18) and data from two participants were not collected for the combustible cigarette condition (n=17).

*Cerebral vascular function*

This was assessed using transcranial Doppler, as previously described^19-23^. Middle cerebral artery (MCA) mean blood velocity was measured using transcranial Doppler ultrasound (TCD) following standard procedures^24^. Briefly, a 2-MHz TCD probe (Neurovision TC, Multigon Industries Inc.; Yonkers, NY) was placed on the left temple, superior to the zygomatic arch, and attached using an adjustable headband to maintain probe placement. Following insonation of the MCA through the transtemporal window, the TCD signal was optimized by adjusting the probe angle and insonation depth, gain, and amplitude. Each participant was then fitted with a mouthpiece attached to a three-way stopcock (Hans Rudolph; Shawnee, KS) that permitted rapid switching between ambient air and a 5 L rubber rebreathing bag (GPC Medical Ltd.; New Delhi, India) pre-filled with the participant’s expired air. Partial end-tidal CO_2_ pressure (P_ET_CO_2_), a surrogate for the partial pressure of arterial CO_2_, was measured continuously through a sampling line connecting the mouthpiece to a capnograph (Capnocheck Plus, Smiths Medical; Dublin, OH). Peripheral oxygen saturation (S_p_O_2_) was monitored throughout the protocol with a digital pulse oximeter (Capnocheck Plus, Smiths Medical; Dublin, OH) placed on a finger. Respiratory excursions were measured with a piezo-electric respiration transducer (Pneumotrace II, UFI; Morro Bay, CA) placed around the abdomen. Due to technical difficulties/loss of follow up, data from three of the participants were not collected for the e-cigarette condition (n=16) and from 6 participants for the combustible cigarette condition (n=13).

*Subjective measures*

The selection of these measures was driven by the suitability for our study’s objectives and the prior use in studies assessing multiple tobacco products^25,26^. Due to loss of follow up, data from two participant were not included (n=17). Using a computer tablet, participants responded to items in two questionnaires.

The modified cigarette evaluation questionnaire (mCEQ)

This is a validated and widely used 11-item questionnaire adapted for e-cigarettes^27^ and answered using a 7-point Likert scale. The mCEQ was administered after each smoking/vaping session. This scale assesses participants’ perception of the following: 1) Satisfying, 2) Taste, 3) Makes you dizzy, 4) Calms you down, 5) Makes you concentrate, 6) Feels more awake, 7) Reduces hunger, 8) Makes you nauseous, 9) Feels less irritable, 10) Enjoying the sensations of smoke in throat and chest, and 11) Reduction of craving.

Duke Sensory Questionnaire (DSQ)

DSQ has nine items: 1) How much did you like the puffs?; 2) How satisfying were the puffs?, 3) How high in nicotine were the puffs?, 4) How similar to your own brand/flavor were the puffs?, and rate the strength of puffs on 5) tongue, 6) nose, 7) back of mouth and throat, 8) windpipe, and 9) chest. Responses were assessed on a 7-point Likert scale (1=not at all; 7=extremely)^28^. The DSQ was administered after each smoking/vaping session.

### Instrumentation

Following the smoking/vaping session, each participant transitioned to an exam table with measurement made in the supine position. Participants were instrumented for the continuous measurement of heart rate, via electrocardiography (CardioCard, Nasiff Associates; Central Square, NY), and intermittent blood pressure, via electrosphygmomanometry (Tango+, SunTech; Raleigh, NC). On the arm contralateral to the intermittent blood pressure cuff, non-invasive, beat-to-beat mean arterial blood pressure was measured via finger photoplethysmography during the rebreathing protocol (MAP; Finometer Pro, Finapres Medical Systems; Netherlands).

### Protocol – peripheral and cerebral vascular function

All peripheral and cerebral vascular data collection was performed by the same operator as were the subsequent analyses of these data. Following instrumentation and a 15 min stabilization period, FMD and RH were measured to assess peripheral vascular function. After a 2 min baseline, during which brachial artery diameter and blood velocity were continuously measured, the pneumatic cuff was inflated to about 220 mmHg for 5 min to elicit ischemia. Upon cuff deflation, brachial artery diameter and blood velocity were recorded for an additional 3 min. Immediately after the FMD/RH assessment, each participant was instrumented as outlined above for the hypercapnic challenge. Each participant breathed ambient air for 3 min for baseline data collection of V_MCA_, MAP, P_ET_CO_2_, S_p_O_2_, heart rate, and respiratory rate. Immediately after this baseline period, each participant performed the rebreathing protocol as previously described^19,20,23^. Briefly, the three-way stopcock Y-valve was switched from ambient air to the rebreathing bag such that the participant expired into and inspired from the 5 L bag, slowly raising P_ET_CO_2_. Rebreathing was continued until the participant reached discomfort, a discernable plateau in P_ET_CO_2_ was established, or 3 minutes elapsed, whichever came first. Following rebreathing, the Y-valve was switched back to ambient air for a 3-min recovery period. Throughout the rebreathe protocol, 100% oxygen was continuously administered into the 5 L bag to maintain arterial normoxia (S_p_O_2_ about 97%)^19-23^

### Data acquisition and analysis

Brachial artery responses following a brief period of forearm ischemia were continuously measured on a second-by-second basis. Brachial artery diameter (D; cm) and mean blood velocity (V_BA_, mean of summed anterograde and retrograde velocities; cm/s) were used to subsequently calculate shear rate (4V_BA_/D; s^-1^) and blood flow [π (D/2)^2^ V_BA_ 60; mL/min]. Peak V_BA_ was identified, as an index of reactive hyperemia and microvascular function, as the highest three-second rolling average value following cuff release. In addition, shear area-under-the-curve (AUC) was calculated as the sum of second-by-second blood shear rate, from the end of occlusion until peak brachial artery diameter^18^.

All data during the hypercapnic challenge were collected using a data acquisition system and software (PowerLab and LabChart 8, ADInstruments; Colorado Springs, CO) and stored on a laboratory computer for offline analysis. All variables were taken as a 1-min average during baseline and on a breath-by-breath basis during the rebreathing protocol. Cerebrovascular conductance index (CVCi) was calculated as V_MCA_/MAP and expressed in both absolute and relative (i.e. percent change from baseline; ΔCVCi) terms. The absolute change in P_ET_CO_2_ (ΔP_ET_CO_2_) was assessed over the entire rebreathing, and MAP and CVCi were recorded as three breath averages at the highest common magnitude of hypercapnia achieved during pre and post data collection sessions within each participant. The relative response was calculated and averaged to determine mean cerebral vascular responsiveness during hypercapnic rebreathing.

All baseline hemodynamic variables and all indices of peripheral vascular and cerebral vascular function were analyzed using GraphPad Prism 9.0 (GraphPad Software Inc., La Jolla, CA) and SPSS 28 (IBM Corp.; Armonk, NY) and presented as mean with standard deviation (SD). Some participants did not complete both conditions due to various reasons including loss of follow-up due to the COVID pandemic. Accordingly, these data were assessed utilizing mixed-effects models with the main effects of condition (e-cigarette vs cigarette) and time (pre and post) as implemented in GraphPad Prism 9.0. The mixed-effects models were run using a compound symmetry covariance matrix and fit using restricted maximum likelihood. Means were calculated for measures of mCEQ and DSQ, and compared using two-tailed paired samples t-tests or Wilcoxon signed-rank test when applicable. Significance was set at α=0.05 and data are presented as mean (SD), unless otherwise noted.

### Sample-size calculation

The sample size was based on the two-tailed paired t-test results from the study of Biondi-Zoccai et al.^8^ which compared brachial artery FMD before and after the single use of three products: heat-not-burn cigarettes, e-cigarettes, and combustion cigarettes^28^. The effect sizes corresponding to those results ranged from 0.7 to 1.3, which are predominantly considered large using Cohen’s criteria^29^. To achieve a power of 0.80 with a significance level of α=0.05, the estimated sample size required, calculated using G*Power 3.1 software (http://www.gpower.hhu.de), was 15 participants.

## RESULTS

Study participants (n=19) had a mean age of 25.2 (SD=5.4) years with range 21–43 years. Participants were mainly males (n=16; 88.8%). On average, participants smoked 6.1 cigarettes per day (SD=1.4). The mean age for starting smoking cigarettes was 18.8 (SD=2.3) years. The mean eCO level at screening was 2.8 ppm (SD=2.5).

Baseline hemodynamic values for heart rate (HR), mean arterial pressure (MAP), and baseline brachial artery diameter, velocity and blood flow were similar prior to the acute vaping and smoking sessions before the flow mediated dilation protocol (p>0.05 for all, Table 1). Following the vaping and smoking sessions all baseline values were also similar between conditions (p>0.05 for all, Table 1). Each value was also similar relative to their respective pre-vaping or pre-smoking values (p>0.05, Table 1), except for HR which was significantly elevated following both the acute vaping and smoking sessions (main effect of time p=0.001).

**Table 1:** Baseline hemodynamic variables (mean ± SD) before and after the acute e-cigarette vaping and cigarette smoking sessions in a crossover laboratory study among adult cigarette smokers

	*E-cigarette*		*Cigarette*		*Interaction p*
	*Pre*	*Post*	*Pre*	*Post*	
HR (bpm)	61 ± 10	69 ± 12*	62 ± 7	67 ± 7*	0.19
MAP (mmHg)	86 ± 7	89 ± 6	88 ± 7	88 ± 8	0.11
P_ET_CO_2_ (mmHg)	44 ± 6	45 ± 4	44 ± 4	44 ± 5	0.34
MCA_Vmean_ (cm/s)	65 ± 15	68 ± 11	62 ± 11	65 ± 10	0.72
CVCi (cm/s per mmHg)	0.76 ± 0.18	0.78 ± 0.13	0.71 ± 0.15	0.75 ± 0.14	0.40
Brachial artery diameter (mm)	3.8 ± 0.6	3.8 ± 0.6	3.7 ± 0.6	3.9 ± 0.6	0.22
Brachial artery V_mean_ (cm/s)	20 ± 11	18 ± 15	20 ± 14	16 ± 11	0.48
Brachial artery flow (mL/min)	128 ± 72	114 ± 78	141 ± 81	119 ± 87	0.77

For e-cigarette vaping session: n=18 for peripheral and n=16 for cerebral vascular measures; while for the cigarette smoking session: n=16 for peripheral and n=13 for cerebral vascular measures. HR: heart rate. MAP: mean arterial pressure. P_ET_CO_2_: end-tidal carbon dioxide tension. MCA_Vmean_: mean blood velocity in the middle cerebral artery. CVCi: cerebral vascular conductance index. V_mean_: mean blood velocity in the brachial artery. Data were analyzed using mixed-effects models.

* Post condition significantly different relative to the pre-condition (main effect of time, p<0.001).

### Peripheral vascular function

**Figure 1:**
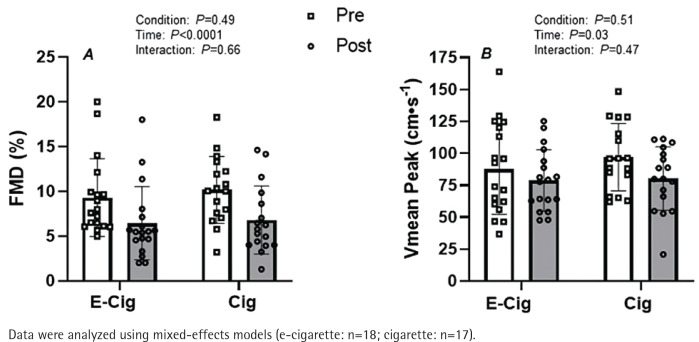
Impact of vaping and smoking on peripheral vascular function in a crossover laboratory study among adult cigarette smokers. A) Brachial artery flow-mediated dilation (FMD), and B) peak blood velocity (Vmean Peak) responses to a brief period of forearm ischemia before (white bars) and after (gray bars) the acute e-cigarette vaping and cigarette smoking sessions

While there were no differences at baseline, the acute vaping and smoking sessions significantly attenuated brachial artery FMD (Figure 1A, main effect of time p<0.001). The magnitude of this reduction in brachial artery FMD was similar between conditions (Figure 1A, condition × time interaction; p=0.66). Likewise, microvascular function as indexed by reactive hyperemia was significantly blunted following both the acute vaping and smoking sessions (Figure 1B; main effect of time p=0.03). However, the magnitude of this reduction was similar between conditions (Figure 1A, condition × time interaction; p=0.47). Accordingly, to account for any potential effects of changes in microvascular function, the FMD response was corrected for changes in shear rate AUC from the end of occlusion until peak brachial artery diameter before and after exposure. The results indicate a main effect of time on this normalized FMD (main effect of time p=0.04, data not shown) with a similar response between conditions (condition × time interaction; p=0.52, data not shown).

### Cerebral vascular function

Baseline hemodynamic values for HR, MAP, MCA_Vmean_, CVCi, and P_ET_CO_2_, were similar prior to the acute vaping and smoking sessions before rebreathing induced hypercapnia (p>0.05 for all, Table 1). Following the vaping and smoking sessions, baseline values (prior to rebreathing induced hypercapnia) were similar between conditions and were also similar relative to their respective pre-vaping or pre-smoking values (p>0.05 for all, Table 1).

**Figure 2:**
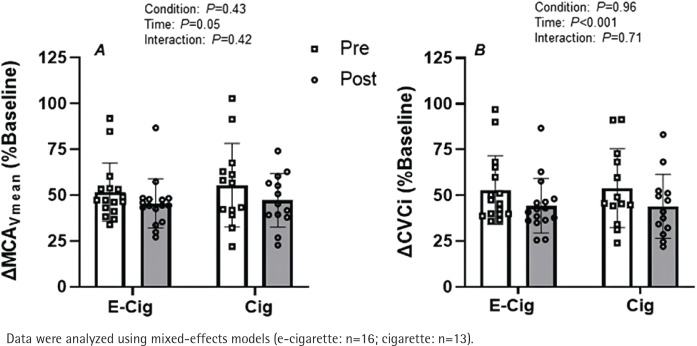
Impact of vaping and smoking on cerebral vascular function in a crossover laboratory study among adult cigarette smokers. A) Relative change in mean blood velocity in the middle cerebral artery (ΔMCA_Vmean_) and B) cerebral vascular conductance index in the middle cerebral artery (ΔCVCi) during the hypercapnic challenge with respect to baseline

Rebreathing induced hypercapnia resulted in an expected increase in P_ET_CO_2_ (change of about 13 mmHg for all conditions; main effect of time, p=0.03), relative to baseline values, following both vaping and smoking sessions. However, the magnitude of rebreathing induced hypercapnia was similar between conditions both before and after the acute exposure sessions (condition × time interaction; p=0.71). The percent increase in MCA_Vmean_ tended to be elevated during hypercapnia before each exposure session with respect to the post-exposure session (Figure 2A; main effect of time p>0.05) with no difference between conditions (Figure 2A, condition × time interaction; p=0.42). When analyzed as CVCi, the results revealed that the magnitude of hypercapnia induced vasodilation was significantly blunted following both the acute vaping and smoking sessions (Figure 2B; main effect of time p<0.01). However, the magnitude of this effect was similar between both conditions (Figure 2B, condition × time interaction; p=0.71).

### Subjective measures

**Figure 3:**
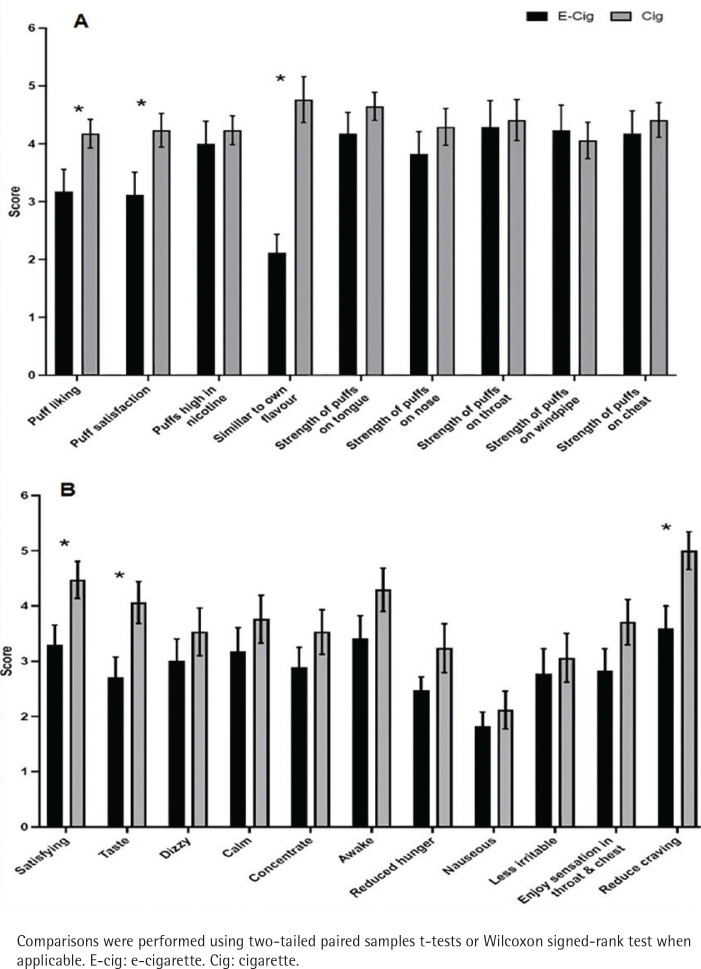
Impact of vaping and smoking on subjective experiences in a crossover laboratory study among adult cigarette smokers. A) Mean ± SEM for post-smoking/vaping subjective experiences using the Duke Sensory Questionnaire (DSQ) among adult smokers (n=17). B) Mean ± SEM for post-smoking/vaping subjective experiences using the Modified Cigarette Evaluation Questionnaire (mCEQ) among adult smokers (n=17)

Subjective responses revealed significant differences between the two conditions (e-cigarette vs cigarette). Figures 3A and 3B depict mean ratings for DSQ and mCEQ, respectively. For the DSQ, significant differences were observed between conditions for the following indices: puff likeness, puff satisfaction, and similarity of product to smoker’s own used brand. For each of these items, values were greater after cigarette smoking (p<0.05 for all) compared with vaping. For the mCEQ, significant differences were observed between the study conditions for the following indices: satisfying product, liking the taste of the product, and reduced craving. For each of these items, values were greater after smoking the combustible cigarette condition compared with vaping (p<0.05 for all).

## DISCUSSION

In this pilot study, we demonstrated that similar to combustible cigarette smoking, exposure to a pod-based ‘next generation’ e-cigarette led to acute impairments in endothelial and cerebral vascular function among adult cigarette smokers. However, vaping a pod-based e-cigarette provided a less satisfactory subjective experience compared to combustible cigarette smoking.

Exposure to an e-cigarette resulted in a marked reduction in endothelium-dependent vasodilation. This impairment in endothelial function was in parallel to the impact observed among the same set of adult smokers following smoking combustible cigarettes. These finding are in agreement with previous studies that used older generations of e-cigarettes. For example, a study by Carnevale et al.^30^ which compared the acute impact of combustible cigarette and e-cigarettes among healthy smokers and non-smokers on endothelial function and on markers of oxidative stress, revealed similar result. The same study showed that acute exposure to both a cigarette and an e-cigarette was shown to increase markers of oxidative stress and to decrease FMD in smokers and non-smokers, with no significant difference between the effects of cigarettes and e-cigarettes^31^. These results are also supported by findings from an animal model study showing that mainstream aerosol from pod-based devices and previous generation e-cigarettes impairs endothelial function in rats, comparable to the impairment caused by cigarette smoke^31,32^. While the study provided an important insight from animal models, to our knowledge, our study is the first to compare the effect of a high nicotine pod-based e-cigarette (JUUL) with that of smoking cigarettes on endothelial function among a sample of regular adult cigarette smokers.

Although the underlying mechanisms of endothelial impairments are multifactorial, elevated production of reactive oxygen species (ROS) and uncoupled endothelial nitric oxide synthase are believed to be the main contributors^33^. It has been shown that cigarette smoking may affect endothelial function through oxidative stress, inflammation and nitric oxide (NO) reduction^30^. The same findings were observed after vaping e-cigarettes which led to a rapid and acute increase in circulating markers of oxidative stress and reduced NO bioavailability^30^. Overall, our findings raise concerns regarding the impact of vaping on cardiovascular health and the potential long-term effects of repeated exposures. In fact, previous studies have shown that sub-clinical inflammation and impairments in vascular function are significantly associated with adverse future cardiovascular events^34,35^.

We also assessed the impact of vaping on cerebral vascular function among adult cigarette smokers. This specific vascular endpoint has been largely overlooked particularly in studies assessing health effects associated with e-cigarette use. Our findings show that vaping resulted in a significant reduction in cerebral vascular function compared with baseline, similar to smoking combustible cigarettes. While our results regarding vaping’s impact on cerebral vascular function followed acute exposure (10 puffs), it is important to consider the possible clinical significance related to chronic vaping on future cerebrovascular events. This is critical given that cerebral vascular function is a predictor and contributor to various cerebrovascular events. Imbalances in cerebral hemodynamics among smokers play a major role in the pathogenesis of stroke, cerebral aneurysm, and increased risk of death^13,36^.

Because of the common claim regarding the potential role of e-cigarette as a substitute for cigarette smoking, we assessed the subjective experiences associated with both vaping an e-cigarette and smoking cigarettes among adult cigarette smokers. Following the combustible cigarette smoking session – that was proceeded by 12 hours of abstinence from any tobacco products – participants rated cigarette smoking as more satisfactory, better tasting, and with better craving suppression compared with vaping e-cigarette. Our finding agrees with Bono et al.^37^ who reported results from two different studies that compared subjective responses among cigarettes smokers after using an e-cigarette and their own brand of combustible cigarettes. Their findings showed that across both studies, the majority of participants rated combustible cigarettes as more satisfying, more pleasant, better tasting, and more calming^37^. Accordingly, our results question the notion of e-cigarettes being a satisfactory alternative to smoking among adult cigarette smokers. However, this is based on a single acute exposure to pod-based e-cigarette vaping among habitual cigarettes smokers that are conditioned to smoking. Therefore, our results should be confirmed in prospective studies assessing the subjective experience of pod-based e-cigarettes vaping (compared with cigarettes) among cigarette smokers over a sustained period of time.

### Strengths and limitations

This study is not without limitations as we did not simultaneously measure plasma nicotine concentrations after smoking a tobacco cigarette or vaping an e-cigarette. Nonetheless, even with a brief exposure protocol, vaping pod-based e-cigarettes resulted in an impairment of peripheral and cerebral vascular function, which is profound given the prolonged and sustained puffing behavior usually manifested by habitual e-cigarette users. Moreover, our assessments of cerebral vascular function were done on young and healthy individuals without any chronic health or psychiatric conditions, and without regular use of prescription medications. It is uncertain whether the findings can be extended to populations at risk or with existing chronic health conditions. Nonetheless, reduced vasodilator responsiveness in the cerebral circulation is present in various populations with impaired vascular function and is a predictor and contributor to cognitive decline, dementia, Alzheimer’s disease, stroke, and increased risk of death^13,27^. Furthermore, because subjective responses to pod-based e-cigarette vaping were reported based on a single acute exposure session among habitual cigarettes smokers who were e-cigarette naïve, these results are preliminary and should be interpreted as such. Similarly, because we focused only on a single exposure session of pod-based e-cigarettes and combustible cigarettes, we did not provide insights on the chronic comparative impact of these products. It is important that future studies extend our findings by assessing whether the chronic use of these products may lead to more persistent vascular impairments. Also, the small sample size in our pilot study might has obscured some significant differences in impairments of peripheral and cerebral vascular function between the two study conditions. Finally, because this is a small-scale pilot study, findings cannot be generalized to the lager population.

Despite these limitations, to our knowledge, this is the first study to assess the impact of vaping high-nicotine-delivery pod-based e-cigarettes on cerebral vascular function among adult cigarette smokers. A major strength of our study is the use of the latest generation e-cigarette device known as JUUL, which is widely popular and delivers high concentration of nicotine efficiently. This represents an improvement over previous studies, which yielded inconclusive findings as a result of utilizing a variety of older generation e-cigarette devices with variable voltages and nicotine concentrations.

## CONCLUSIONS

Our pilot study indicates that, similar to smoking a cigarette, vaping pod-based e-cigarettes leads to an impairment in endothelial function and cerebral vascular function among cigarette smokers while providing a less satisfactory subjective experience compared with cigarettes among regular adult smokers. Accordingly, our pilot study suggests that pod-based e-cigarettes vaping may neither be a safe nor satisfactory alternative to cigarette smoking. Therefore, further longitudinal studies are needed to assess the long-term impact of high-nicotine-delivery pod-based e-cigarette use on cardiovascular function compared with combustible cigarettes.

## Data Availability

The data supporting this research are available from the authors on reasonable request.
